# Health-related quality of life scores after intensive care are almost equal to those of the normal population: a multicenter observational study

**DOI:** 10.1186/cc13059

**Published:** 2013-10-13

**Authors:** Lotti Orwelius, Mats Fredrikson, Margareta Kristenson, Sten Walther, Folke Sjöberg

**Affiliations:** 1 Departments of Intensive Care, Linköping University/County Council, Linköping, Sweden; 2 Clinical and Experimental Medicine, Linköping University/County Council, Linköping, Sweden; 3 Medicine and Health Sciences, Linköping University/County Council, Linköping, Sweden; 4 Burns, Hand and Plastic Surgery, Faculty of Health Sciences, Linköping University/County Council, Linköping, Sweden

## Abstract

**Introduction:**

Health-related quality of life (HRQoL) in patients treated in intensive care has been reported to be lower compared with age- and sex-adjusted control groups. Our aim was to test whether stratifying for coexisting conditions would reduce observed differences in HRQoL between patients treated in the ICU and a control group from the normal population. We also wanted to characterize the ICU patients with the lowest HRQoL within these strata.

**Methods:**

We did a cross-sectional comparison of scores of the short-form health survey (SF-36) questionnaire in a multicenter study of patients treated in the ICU (*n* = 780) and those from a local public health survey (*n* = 6,093). Analyses were in both groups adjusted for age and sex, and data stratified for coexisting conditions. Within each stratum, patients with low scores (below -2 SD of the control group) were identified and characterized.

**Results:**

After adjustment, there were minor and insignificant differences in mean SF-36 scores between patients and controls. Eight (*n* = 18) and 22% (*n* = 51) of the patients had low scores (-2 SD of the control group) in the physical and mental dimensions of SF-36, respectively. Patients with low scores were usually male, single, on sick leave before admission to critical care, and survived a shorter time after being in ICU.

**Conclusions:**

After adjusting for age, sex, and coexisting conditions, mean HRQoL scores were almost equal in patients and controls. Up to 22% (*n* = 51) of the patients had, however, a poor quality of life as compared with the controls (-2 SD). This group, which more often consisted of single men, individuals who were on sick leave before admission to the ICU, had an increased mortality after ICU. This group should be a target for future support.

## Introduction

Health-related quality of life (HRQoL) has been reported to be significantly worse among patients who have been treated in intensive care units (ICUs) than among the general population [1,2]. Many factors have been claimed to have important effects on this, usually ICU related [1]. Recently increasing evidence supports the idea that coexisting medical conditions may be important, and possibly the main factor in reducing HRQoL among patients who have been treated in an ICU [3,4]. It must then be appreciated that older age and the presence of coexisting conditions are more common among patients who have been treated in ICUs [3,5-7] than in their control groups. Although many patients in ICU have one other disease, the relative contributions among those with more than one condition is considerable [3].

Control groups, in these studies, usually comprised patients either in hospital or living at home. The latter group is usually used, and they are more often young and with few coexisting conditions [4]. The high incidence of coexisting conditions in patients in ICUs raises the question of how these can influence the evaluation of HRQoL after critical care. More precisely, what is the magnitude of the difference in HRQoL in patients previously treated in ICU compared with that of a control group also stratified for coexisting disease? Such a control group has not, as far as we know, been used in such an evaluation.

Our aim was therefore to compare the HRQoL of patients who had been in the ICU with that of a reference group gathered from the uptake areas of the hospitals participating in the study and, in addition to adjusting data for age and sex, also to stratify for coexisting disease. Our hypothesis was that after such stratification, the magnitude of the difference in HRQoL between ICU patients and controls would be less than previously shown. As also the general population has a wide variation in HRQoL, with SF-36 scores ranging from 0 to 100, we *a priori* also defined the lower limit of normal HRQoL at -2 standard deviations (SDs) below the mean SF-36 score of the comorbidity stratified value of the age- and sex-adjusted control group (30).

In addition, we wanted to identify the proportion of patients with poor HRQoLs and to assess their characteristics.

In accordance with our prestudy hypothesis, our main findings were; first, the differences in HRQoL between the ICU patients and the general reference group were significantly decreased after adjusting for age and sex, and stratified for coexisting disease. Second, up to 22% of the patients had an HRQoL level below the reference group (-2 SD). Last, risk factors for HRQoL below the reference group were being single and male, having a sick leave before ICU admission, and increased mortality early after ICU.

## Material and methods

We used a cross-sectional analysis to compare data from a multicenter study of patients who had been in an ICU in the southeast of Sweden, the “Livskvalitet Intensivvård” (LIVA) study [3], with data from a control group from a public health survey of a normal population [8].

### Study group

Data from former ICU patients were collected in the LIVA study, from 2000, and completed during 2004. The aim of that study was to measure and describe HRQoL among former patients from the ICU after discharge from hospital. These results have been published both in the short term (6 months) [4], for separate diagnoses such as trauma [9], and longitudinally (up to 3 years) [3]. In the previous studies, comorbidity stratification has not been performed, in either the study group or in the controls.

The ICUs included in the study were in one university and two general hospitals, each of which admitted 500 to 750 ICU patients annually. Of these admissions during the study, 93% were emergencies, and the most common primary diagnoses were sepsis, multiple trauma, and disturbances in the respiratory or circulatory systems, or both. Patients with primary coronary disease, those recovering from heart surgery, neurosurgical patients, neonates, and patients with burns are treated at other specialized units and were excluded.

All patients aged between 20 and 74 years who were admitted consecutively between 1 August 2000 and 30 June 2004, who remained in the ICU for more than 24 hours, who were alive 6 months after discharge from hospital, and who consented to participate in the study, were included. The upper age limit was chosen to match the age of the control group (20 to 74 years). Patients who were readmitted were included only on their first admission. The national Swedish Social Security register was checked to avoid sending inquiries to patients who had died. Six months after discharge from the ICU, we sent information and a request to participate to each patient by mail, together with a structured questionnaire and a preaddressed and prepaid envelope. Patients who had not responded within about 10 days were contacted by telephone by one of the investigators (LO). If telephone or first mailing achieved no answer, two reminders were sent (at 3 and 6 weeks).

All admissions to ICU at all hospitals are recorded electronically in databases. From these databases, data were extracted about patients’ sex, age, reasons for admission to and duration of stay in ICU, APACHE II score on admission, and outcome (dead or alive).

### Control group

Data from a questionnaire-based public health survey of the population of the county of Östergötland (the area in which the university hospital is situated and adjacent to the county where the general hospital is located) were used for the reference group [8]. That survey aimed at monitoring health and health-related risk factors in the population and was completed during the year 1999. Questionnaires were mailed to a random sample of 10,00 people aged 20 to 74 years. After two reminders, 6,093 (61%) had responded. Apart from lower percentages of immigrants and single households, the responders differed only marginally from the reference population of the county [8].

The study was approved by the Committee for Ethical Research at the University of Health in Linköping.

### Questionnaires and instruments

In both datasets, the instrument chosen for the evaluation of HRQoL was the medical outcome Short Form health survey (SF-36) version 1 [10]. SF-36 is well known internationally and has been recommended for measuring HRQoL in critical care [11]. It is reliable and valid for use in the ICU [12,13] and has been translated into Swedish and validated in a representative sample [14]. It has 36 questions and generates a health profile of eight subscales: physical functioning (PF), limitation of the role by physical problems (RP), bodily pain (BP), general health (GH), vitality (VT), social functioning (SF), limitation of the role by emotional problems (RE), and mental health (MH) [14]. The scores on all the subscales are transformed to a scale from 0 = worst to 100 = best [15].

In both groups, coexisting conditions were assessed from self-reports of present disease, as previously described [3,4,9,16]. In the ICU questionnaire, the questionnaire asked, “Do you have any of the following diseases and have had it for more than 6 months prior to admission to the ICU?” with the prespecified alternatives of cancer; diabetes; heart failure; asthma/allergy; rheumatic, gastrointestinal, blood, kidney, psychiatric, or neurologic disease; thyroid or any other metabolic disturbance; or other long-term disease?” Information was also collected about participants’ civil status, employment before and after admission to the ICU, educational level, and self-reported sick leave before they were admitted to the ICU.

The control-group questionnaire included, apart from questions on background characteristics, a wide array of questions about health problems. These questions assessed the frequency (daily, weekly, monthly, and rarely/never) of specific symptoms of ill health during the previous 12 months, some of which were specific to certain health problems and diseases such as asthma and cardiovascular disease. Also, this section of the survey was concluded with an open question that asked about “other health problems.” Although different from the questions used to assess preexisting disease in the ICU patients, the questions on health problems made it possible to classify the reference population into disease groups corresponding to those reported by the ICU patients. This was done in the following way. One of the authors (MK, MD) transformed the free-text information regarding such “other health problems,” which were basically of two categories; one was Latin names of diagnoses, which were well defined; the other was, to a large extent, symptoms, from the International Classification of Diseases-10 nomenclature. Milder symptoms (low intensity and infrequent) were overlooked. Classification of the reference group into disease-specific subgroups was based on symptoms reported as daily or weekly on one or more questions within the same disease category, or the International Classification of Diseases-10 labels put on the “other health problems” reported. As no questions about symptoms of cancer and diabetes were included in the questionnaire, the cancer and diabetes subgroups were based solely on the second question, whereas the other disease-specific subgroups (cardiovascular disease, gastrointestinal disease, and asthma) were based on either one, or a combination of the two. In the majority of cases, the classification of respondents having a disease was based only on the information from the open-ended question (mainly Latin diagnoses).

### Statistical analysis

Descriptive data are presented as mean (SD), range, and percentage. The patients and the control group were each divided into three groups for coexisting conditions/other conditions: those who had previously been healthy (that is, they had had no disease before they were admitted to the ICU (for the reference groups, no disease)), those who had one coexisting disease, and those who had more than one.

Within each stratum, models were built on a linear regression analysis for the control group. Scores for each dimension of SF-36 were used as dependent variables one at a time, and the independent variables were age and sex, according to general conventions. The specified model was then applied to the patient’s model, and a predicted dimension was calculated based on the patient’s age and sex. In the model, we used the mean value for each of the eight SF-36 dimensions adjusted for age, sex, and coexisting condition.

The predicted values for patients were then compared with the -2 SD value for the control group, for each stratum. Those patients who had lower predicted values than the limit for the controls were considered to be outside the “reference range.”

Probabilities of <0.05 were accepted as significant. Statistical analyses were aided by the use of the Statistical Package for the Social Sciences (SPSS, named PASW from version 18.0; Chicago, IL, USA).

## Results

**Figure 1:**
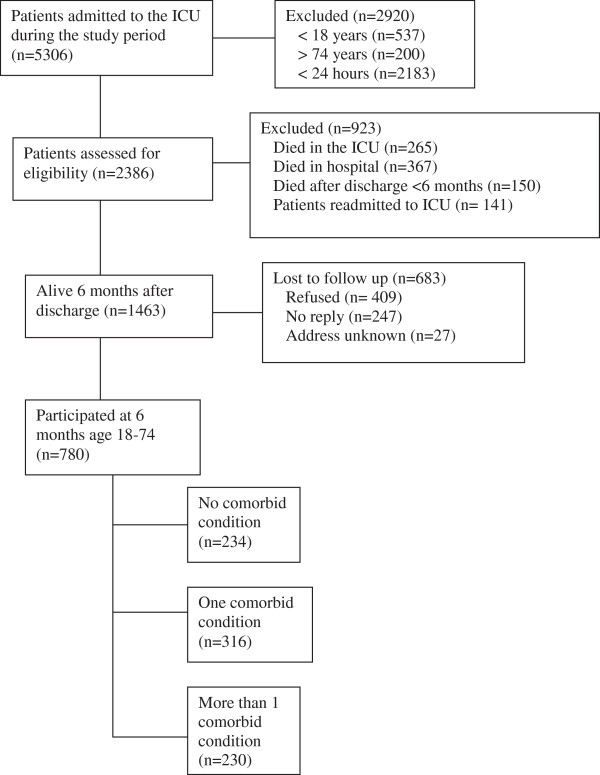
Outline of the protocol of the study.

Of the 5,306 patients admitted during the study period, 780 were eventually included in this study. The reasons for exclusions and number of patients in the three groups are shown in the flow chart (Figure 1). The proportion of patients discharged alive from ICU who were alive at 6 months after ICU care and who did provide a response was 53%. Clinical and personal details of the patients are shown in Table 1 and, for the control group, in Table 2.

**Table 1:** Details of patients studied

	**Total study group**		**No comorbidity**		**Comorbidity 1 disease**		**Comorbidity >1 disease**	
	**(*n*** **= 780)**	**Range**	**(*n*** **= 234)**	**Range**	**(*n*** **= 316)**	**Range**	**(*n*** **= 230)**	**Range**
No of men/women (%)	444/336 (56.9)		131/103 (56.0)		181/135 (57.3)		132/98 (57.4)	
Age (years)	46.4 (15.8)	52.5 (15.8)	48.0 (17.3)	18–74	52.9 (15.2)	19–74	56.4 (14.0)	19–74
APACHE II score	14.8 (7.6)	0-43	12.7 (7.1)	0–34	15.2 (7.5)	0–42	16.6 (7.9)	0–43
Stay in ICU (hours)	129.9 (178.6)	24–1845	100.9 (112.2)	24–665	135.3 (182.2)	24–1,170	152.0 (220.5)	24–1,845
Stay in hospital (days)	14.8 (20.3)	1–231	11.9 (14.4)	1–115	15.4 (19.6)	1–124	16.9 (25.4)	1–231
Time on ventilator (hours)	66.6 (158.6)	0–1753	38.8 (96.9)	0–625	69.0-152.0	0–1,054	91.6 (207.1)	0–1,753
Disease (%)								
Cancer	86 (11.0)							
Diabetes	102 (13.1)							
Cardiovascular	131 (16.8)							
Gastrointestinal	89 (11.4)							
Miscellaneous	523 (67.1)							
No. of diseases (%)								
0	234 (30)							
1	316 (40.5)							
>1	230 (29.5)							

APACHE II, Acute Physiology and Chronic health Evaluation; ICU, Intensive Care Unit.

Data are expressed as mean (SD) or N (%), unless otherwise stated.

**Table 2:** Clinical details of the control group

	**Total study group**		**No comorbidity**		**Comorbidity 1 disease**		**Comorbidity >1 disease**	
	**(*n*** **= 6093)**	**Range**	**(*n*** **= 4386)**	**Range**	**(*n*** **= 1411)**	**Range**	**(*n*** **= 296)**	**Range**
No of men/women (%)	2,822/3,271 (46.3)		2,067/2,319 (47.1)		630/781 (44.6)		125/171 (42.2)	
Mean (SD) age	52.5 (15.1)	46.4 (15.1)	44.7 (15.0)	20–74	49.8 (14.7)	20–74	54.7 (13.1)	20–74
Disease (%)								
Cancer	32 (0.5)							
Diabetes	95 (1.6)							
Cardiovascular	810 (13.3)							
Gastrointestinal	306 (5.0)							
Miscellaneous	1,968 (32.3)							
No. of diseases (%)								
0	4,386 (72.0)							
1	1,411 (23.2)							
>1	296 (4.9)							

The group who did not respond at all in the study (*n* = 683) differed from the group who responded, in that there were fewer men (*P* = 0.02), higher average APACHE II score (*P* = 0.04), shorter LoS in the ICU (*P* < 0.001), shorter time on ventilator (*P* < 0.001), and fewer of the following admission diagnoses to ICU (multitrauma, sepsis, and gastrointestinal disease *P* < 0.001).

The mean age of the patients from ICU was lower (46 years; SD, 15.8) than that of the controls (53 years; SD, 15.1) (*P* < 0.001). There was also a significant sex difference, with more men in the ICU group (57%, *n* = 444) than in the control group (46%, *n* =2,8882) (*P* < 0.001). The prevalence of coexisting/present disease was higher among the ICU patients than the control group, being 70% (*n* = 546) and 28% (*n* = 1,707), respectively (*P* < 0.001). This was also the case for prevalence of more than one disease; patients *n* = 230, 30%; control group *n* = 296, 5% (data not shown).

### SF-36 scores for patients from ICU and the control group

**Figure 2:**
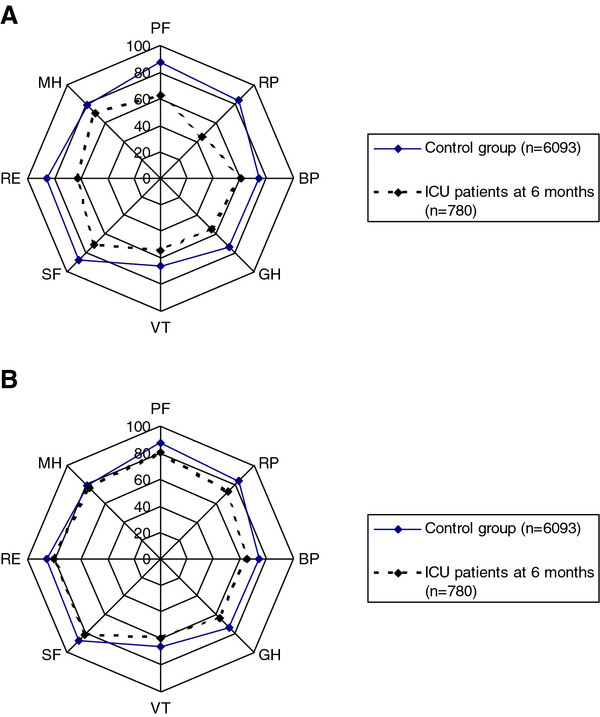
**Health-related quality-of-life differences between the reference group and ICU patients not adjusted for age and sex respectively adjusted for age and sex. (A)** Health-related quality of life (SF-36) values shown for each dimension: physical functioning (PF), role limited by physical problems (RP), bodily pain (BP), general health (GH), vitality (VT), social functioning (SF), role limited by emotional problems (RE), and mental health (MH) in the Polar Plot Chart for the reference group (*n* = 6,093) and patients from ICU (*n* = 780), not adjusted for age and sex. **(B)** Health-related quality of life (SF-36) values shown for each dimension: physical functioning (PF), role limited by physical problems (RP), bodily pain (BP), general health (GH), vitality (VT), social functioning (SF), role limited by emotional problems (RE), and mental health (MH) in the Polar Plot Chart for the reference group (*n* = 6093) and patients from the ICU (*n* = 780), adjusted for age and sex.

SF-36 scores for the whole ICU group and the control group are shown in Figure 2A. The differences between the two groups in all eight dimensions were large and significant (*P* < 0.001 in each case).

**Figure 3:**
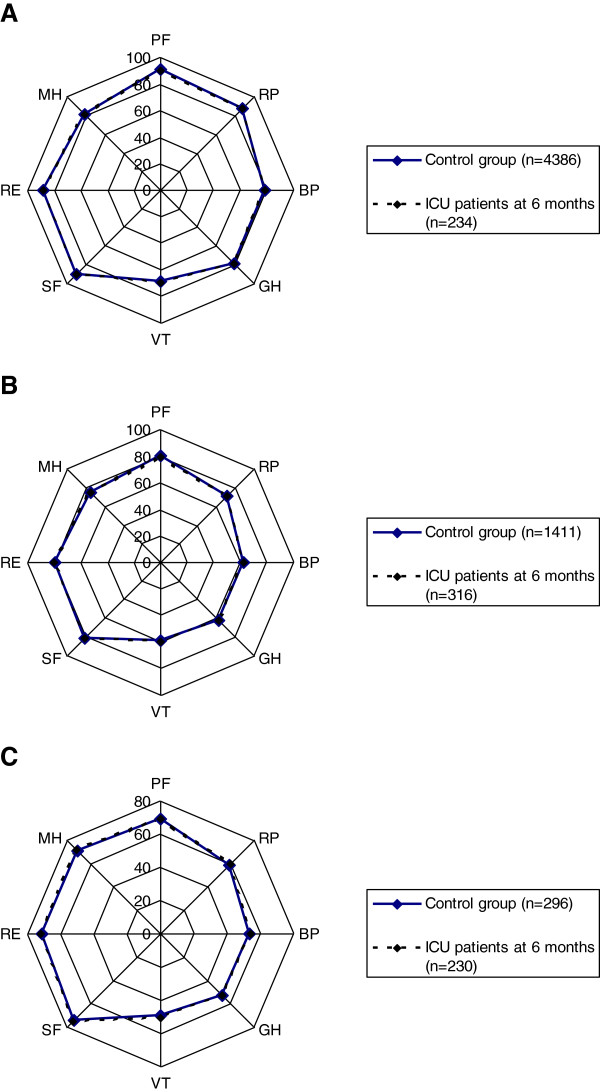
**Health-related quality-of-life differences between the reference group and ICU patients without preexisting diseases, with one preexisting disease, and more than one preexisting disease, respectively. (A)** Health-related quality of life (SF-36) values shown for each dimension: physical functioning (PF), role limited by physical problems (RP), bodily pain (BP), general health (GH), vitality (VT), social functioning (SF), role limited by emotional problems (RE), and mental health (MH) in the Polar Plot Chart for the healthy reference group (*n* = 4,386) and the previously healthy ICU patients (*n* = 234), adjusted for age and sex. **(B)** Health-related quality of life (SF-36) values shown for each dimension: physical functioning (PF), role limited by physical problems (RP), bodily pain (BP), general health (GH), vitality (VT), social functioning (SF), role limited by emotional problems (RE), and mental health (MH) in the Polar Plot Chart for the reference group (*n* = 1,411), and the patients from ICU (*n* = 316) with one preexisting disease, adjusted for age and sex. **(C)** Health-related quality of life (SF-36) values shown for each dimension: physical functioning (PF), role limited by physical problems (RP), bodily pain (BP), general health (GH), vitality (VT), social functioning (SF), role limited by emotional problems (RE), and mental health (MH) in the Polar Plot Chart for the reference group (*n* = 296) and the patients from ICU (*n* = 230) with more than one preexisting disease, adjusted for age and sex.

In Figure 2B, the SF-36 scores for the patients have been adjusted for age and sex, which leads to a pronounced reduction in the differences from the control group in all dimensions. When stratified for the number of coexisting conditions (healthy, one disease, and more than one disease), the remaining differences are further diminished, and the small differences recorded between the patients and the control groups are not significant for any dimension in any of the strata (Figure 3A-C).

### Patients with low SF-36 scores

In Table 3, the number and percentages of patients who had scores below a limit regarded as low (-2 SD of the reference group) are shown for the patients with no coexisting conditions (*n* = 234), one coexisting condition (*n* = 316), and more than one coexisting condition (*n* = 230). The largest percentage of low scores in the physical dimensions was 8% (*n* = 18) and was seen for physical functioning in the strata with no coexisting condition. In the psychosocial dimensions (vitality, social functioning, emotional problems, and mental health), the highest percentage of low scores was 22% (*n* = 51); this was seen for emotional role function in the stratum with more than one coexisting condition.

**Table 3:** Proportions of the ICU patients with SF-36 score 2 SD below the reference group lower level, adjusted for age and sex

	**Number (%)**	**Mean**	**Range**
**Without comorbidity (*n*** **= 234)**			
Physical function	18 (8.0)	27.6	6.9–39.1
Role functioning-physical	0		
Bodily pain	2 (0.8)	9.2	5.9–12.5
General health	6 (2.6)	22.1	11.0–31.8
Vitality	19 (8.1)	9.9	-13.9–19.6
Social functioning	19 (8.1)	26.7	-2.47–37.5
Role function-emotional	31 (13.2)	-5.7	-7.77– –2.10
Mental health	14 (6.0)	23.1	-2.69–35.2
**Comorbidity 1 disease ( *n * = 316)**			
Physical function	5 (1.6)	12.5	10.3–13.4
Role functioning-physical	0		
Bodily pain	10 (3.2)	4.64	-3.06–10.5
General health	2 (0.6)	10.5	6.67–14.4
Vitality	23 (7.3)	-3.65	-13.3–4.40
Social functioning	34 (10.8)	14.4	-4.33–25.0
Role function-emotional	40 (12.6)	-1.72	-1.98– –1.59
Mental health	24 (7.6)	11.5	-6.06–19.6
**Comorbidity >1 disease ( *n * = 230)**			
Physical function	0		
Role functioning-physical	7 (3.0)	-4.06	-9.32– –0.58
Bodily pain	11 (4.8)	5.75	0.70–9.60
General health	0		
Vitality	15 (6.5)	-1.87	-7.48–2.59
Social functioning	25 (10.9)	6.22	-4.62–11.7
Role function-emotional	51 (22.2)	-3.83	-5.10– –3.14
Mental health	28 (12.2)	6.71	-17.8–24.0

Mean and range are data for the values of SF-36 scores of the patients that were found to be below the -2 SD level of the reference group.

### Characteristics of patients with low (-2 SD) SF-36 scores

Compared with other patients, those with low SF-36 scores were more likely to be male, single, and on sick leave before admission to the ICU (*P* < 0.001) (Tables 4 and 5), and had more than a double mortality risk soon after discharge from the ICU (6 months to 3 years) (Table 6). Besides these mortality-dependent changes, we found no effects related to social factors, such as marital state, employment, and education between patients who died within the time period 6 months to 3 years and who had either a low or normal level of HRQoL (Table 7).

**Table 4:** ICU patients with normal SF-36 scores compared with patients with SF-36 score 2 SD below the reference group lower level in at least one of the SF-36 eight dimensions for the total group and divided in those with low level and with or without comorbidity

	**Patients with normal level**	**Patients with low level**		**Without comorbidity**	**Patients with low level**		
	**(*n*** **= 553)**	**(*n*** **= 227)**	***P *** **value**	**(*n*** **= 61)**	**one disease (*n*** **= 81)**	**>1 disease (*n*** **= 85)**	***P *** **value**
Age (years)	53 (16.1)	52 (15.2)	0.468	49 (16.1)	51 (14.8)	54 (14.6)	
No (%) male	284 (51.4)	160 (70.5)	<0.001	42 (69)	59 (73)	22 (27)	0.843
No (%) female)	269 (48.6)	67 (29.5)		19 (31)	59 (69)	26 (31)	
APACHE II score	14.9 (7.6)	14.8 (7.6)	0.939	12.6 (6.2)	14.6 (7.2)	16.6 (8.5)	
Stay in ICU (hours)	125.2 (173.7)	141.5 (189.9)	0.246	117.8 (111.7)	147.5 (175.3)	152.8 (241.3)	
Stay in hospital (days)	14.3 (19.5)	15.8 (22.1)	0.363	13.9 (16.6)	15.7 (20.4)	17.3 (26.6)	
Time on ventilator (hours)	65.3 (156.7)	69.8 (163.5)	0.722	61.7 (128.2)	58.5 (105.0)	86.3 (222.1)	
No (%) deaths during the study	38 (6.9)	38 (16.7)	<0.001	6 (9.8)	10 (12.3)	22 (25.9)	0.016

Data are expressed as mean (SD) unless otherwise stated.

All differences in the table between the patients with low level without comorbidity compared with those with low level and one disease are not significant.

All differences in the table between the patients with low level without comorbidity compared with those with low level and more than one disease are not significant, except for age (*P* = 0.035), and for APACHE (*P* = 0.002).

All differences in the table between the patients with low level and one comorbidity compared with those with low level and more than one disease are not significant.

**Table 5:** Descriptive data for the ICU patients with SF-36 score 2 SD under the reference group lower level in at least one of the SF-36 eight dimensions in comparison with the patients with normal SF-36 scores

	**Patients with normal level**	**Patients with low level**		**Patients with low level**	**Patients with low level**	
	**(*n*** **= 553)**	**(*n*** **= 227)**	***P *** **value**^***b***^	**Died during the study (*n*** **= 38)**	**Survived (*n*** **= 189)**	**P value**^***c***^
Marital state^*a*^			0.004			0.694
Married/cohabit	372 (68.1)	124 (54.6)		22 (59.5)	102 (54.8)	
Single	146 (26.7)	85 (37.4)		12 (32.4)	73 (39.2)	
Widow/widower	28 (5.1)	14 (6.2)		3 (8.1)	11 (5.9)	
Employment before ICU^*a*^			0.456			0.252
Employed/leader	261 (49.1)	106 (46.7)		12 (36.4)	94 (53.1)	
Retired	222 (41.7)	75 (33.0)		17 (51.5)	58 (32.8)	
Other	49 (9.3)	29 (12.7)		4 (12.1)	25 (14.2)	
Sick leave before ICU	63 (11.4)	46 (20.3)	0.001			0.144
Born in Sweden^*a*^	504 (91.3)	199 (87.7)	0.163	35 (92.1)	164 (87.2)	0.398
Education						
Higher than compulsory school^*a*^	353 (64.4)	146 (65.2)	0.841	27 (71.1)	119 (64.0)	0.404
High School/university	131 (24.0)	56 (24.7)	0.841	10 (26.3)	46 (24.3)	0.796

Data are expressed as number (%).

^*a*^Not all patients answered the question.

^*b*^χ^2^test between the patients with low level on SF-36 and the patients with normal level on SF-36.

^*c*^χ^2^test between those who died and the survivors in the group with low level on SF-36.

**Table 6:** **Mortality for the total ICU group (*n*** **= 780)**

	**Dead**	**Survived**	***P *** **value**
With lower SF-36 level than the reference group, total	38 (16.7)	189 (83.3)	<0.001
Dead between 6–12 months	9 (4.0)		
Dead between 13–24 months	18 (7.9)		
Dead between 25–36 months	11 (4.8)		
With normal SF36 level, total	38 (6.9)	515 (93.1)	
Dead between 6–12 months	11 (2.0)		
Dead between 13–24 months	11 (2.0)		
Dead between 25–36 months	16 (2.9)		

Data are expressed as number (%). Chi-Square test between the patients with low level on SF-36 and the patients with normal level on SF-36 in the total groups.

**Table 7:** Descriptive data for the ICU patients who died during the study period with SF-36 score 2 SD under the reference group lower level in at least one of the SF-36 eight dimensions versus those with normal SF-36 score

	**Patients with lower level (*n*** **= 38)**	**Patients with normal level (*n*** **= 38)**	***P *** **value**
Marital state^*a*^			0.490
Married/cohabit	22 (59.5)	22 (59.5)	
Single	12 (32.4)	9 (24.3)	
Widow/widower	3 (8.1)	6 (16.2)	
Employment before ICU^*a*^			0.348
Employed/leader	12 (36.4)	10 (26.3)	
Retired	17 (51.5)	27 (71.1)	
Other	4 (12.1)	1 (2.6)	
Sick leave before ICU	11 (28.9)	2 (85.3)	0.06
Born in Sweden^*a*^	35 (92.1)	33 (89.2)	0.664
Education			
Compulsory school^*a*^	27 (71.1)	22 (57.9)	0.231
High school/university	10 (26.3)	11 (29.7)	0.742

Data are expressed as number (%).

^*a*^Not all patients answered the question.

## Discussion

Several new and important findings resulted from this study.

First, and in line with our starting hypothesis, we found that after stratifying for coexisting disease, no differences were found in HRQoL between patients who had been in an ICU and those in a control group matched for age and sex [1,2]. These conclusions are supported by our previous findings that, after adjusting for coexisting conditions, only minor effects on HRQoL could be related to specific events in ICU, such as high APACHE II score, longer duration of stay, and time spent on a ventilator [3].

Second, and of particular importance for this study, is that after adjusting for age and sex, we used the lower -2 SD cut-off value of the control group (HRQoL) as the lower norm for HRQoL for each of the groups (healthy, one disease, and more than one disease). To our knowledge, this has not been attempted before for ICU-related research. In doing so, we aimed to find the lower reference limit of HRQoL of people in Swedish society with whom to compare the patients who had been in an ICU. By assessing this limit, we aimed to study those patients who constitute the group that have a problem related to HRQoL.

Third, 6 months after discharge from ICU, up to 22% (*n* = 51) of the patients have a perceived HRQoL lower than the worst -2 SD of a normal population. These patients were more likely to be male, single, have been on sick leave before they were admitted to the ICU, but also survived a shorter time (6 months to 3 years) after their stay in the ICU. As this is an important finding, it underlines that these patients should be identified and the underlying factors further explored. The result that their decline was mainly recorded in the mental-dimensions contrasts with findings in previous studies in which the effects have mainly been recorded for the physical dimensions [2,17,18] but may be in line with symptoms related to posttraumatic stress.

Fourth, a strength of this study is that the people studied in both groups are representative of general Swedish conditions [3,19]. For the patients in ICUs, personal and ICU-related variables were also similar to those described in data from the Swedish Intensive Care Registry and in Scandinavia as a whole (ICU population) [20].

Fifth, our approach has led us to conclusions that differ from those of other articles on this topic, the most important of which is that both the magnitude of differences in mean HRQoL and the number of patients who have, substantially, reduced HRQoLs seem smaller than previously shown [3,6,21-25]. The reason for this is mainly the high rate of coexisting conditions among the patients who had been in ICUs compared with that in the control group; not only is the rate of coexisting diseases higher, but the rate of several coexisting conditions within each patient in the ICU is higher. Significant age and sex differences exist between patients who were in ICU and in the control group, and a rather wide “normal” distribution of HRQoL scores appears in the control group that is seldom fully accounted for in critical care research.

Sixth, also an important finding in the study is that not only the effect of comorbidity affects the HRQoL registered after ICU but a significant portion of the patients that actually report a low HRQoL die in the short time after ICU care. Also the largest numbers of deaths are seen before our first measurement at 6 months after ICU discharge and the HRQoL may be assumed to be significantly affected, although they were not studied. Interestingly, we also found that for the study population that died more than 6 months after ICU (6 to 36 months) had a low HRQoL after ICU care, but they constituted a minority. Furthermore, for this group, only minor effects could be related to psychosocial factors. After stratifying the remaining patients for comorbidity, these patients reported an HRQoL close to that of the controls.

Several important issues about the methods that underlie the analysis have implications for the conclusions. The new approaches are that first, we used a control group gathered from or near the uptake areas of the hospitals from which the patients were recruited, and the coexisting conditions in the control group were assessed in the same way (self-reports of present disease) and during a similar time period as the HRQoL data [4]. Although this approach has been used and presented by us before [3,4,16], what is new is that we have divided the reference group, depending on the number of coexisting conditions into those who are healthy, and those who have one or more other conditions. This is a better adjustment for the differences in coexisting comorbidity burden between groups, but unfortunately, it does not compensate for differences between type and severity of diseases, which would be better and should be the aim in future studies.

### Limitations

In evaluating the HRQoL of a group of patients from ICUs, which group is to be used for comparison to assess the effects of ICU care properly? This is debatable. Several research workers have used the patients from ICUs themselves, and their next of kin. In this setting, patients have been asked to estimate their HRQoL before they were admitted to the ICU. Although this approach is logistically appealing, it has been claimed to overestimate the HRQoL before admission to ICU, and lead to higher changes in estimates of HRQoL after discharge [5,21]. It has also been shown that the next of kin underestimate the mental, and overestimate the physical, health of the patient before admission [26,27]. Our use of a control group from the normal population, may at least partly explain the altered [5,7,28].

### Conclusions

Concerning HRQoL outcome data that we have presented, one limitation, however, is that the ICU population had to recall their preexisting diseases in a period of more than 6 months before, whereas the control group did so at the time of filling out the questionnaire. This may be a factor that may increase (patient mistakes the ICU disease as a comorbid state) or decrease, as there might be too few comorbid states listed because of memory difficulties. Both effects are difficult to evaluate. Further, another limitation is that significant disease-specific symptoms that were present besides those that can be traced to the regular ICD coding were converted to ICD diagnoses by one of the authors (MK), but this was performed in a small number of patients (<5%) only.

Another technique is to use control groups, and groups of patients have been approached. Although theoretically promising, this solution often lacks the heterogeneity of patients in the ICU by comprising only certain diagnoses and not including emergency care. With this in mind, a control group based on the general population from the areas of the local hospitals may be advantageous. The shortcoming of such a control group is that it has lower rates of coexisting conditions, possibly a different comorbidity mix, and different age and sex profiles [3]. These factors may have affected the conclusions drawn from this study and, although adjustments were made for age and sex and stratification for coexisting conditions, we lack a disease-specific adjustment, which is a significant shortcoming. Other factors such as psychosocial issues (for example, abuse) that were not adjusted for, may also affect the results and should be addressed as well for a more complete comparison. However, these factors have previously been found less important in the regression model of our previous studies [3].

Yet another important issue about the control group is how to establish what a normal HRQoL is. The figure of -2 SD is often used in other clinical settings, such as within clinical chemistry, and recently also in psychological research [29-31]. However, we have been unable to find any previous publications that have used this approach in HRQoL research related to ICU care. It may also be argued that nonparametric statistical analysis should be used. However, in HRQoL research with large groups of patients (as in the present study), the tradition is to use parametric statistics [15,32]. We think both these approaches may affect the results, but related to the extensive previous use of these techniques, such effects may be claimed to be minor.

A further point of contention is that, in the present study, an approach by stratifying for the number of diseases (healthy, one other disease, and more than one other disease) does not take into account the specific effects of each of the different diseases. A better approach would then be to adjust for, say, type of disease. Although such an adjustment has recently been made for HRQoL, this approach has not been evaluated in Europe, and we therefore did not use it in this study [29]. It would also be preferable to adjust not only for the specific disease but also for the severity of each disease. This can be done for certain conditions such as heart failure and malignant disease [30,31], but is difficult in the ICU with a heterogeneous population.

We did not take into account the fact that some patients obtain a diagnosis of chronic disease during their stay in ICU or during the follow-up period, and this should also have been adjusted for. This would affect mainly the evaluation of the previously healthy patients in the ICU or those with one or more diseases. They would be considered to have HRQoL that was falsely reduced after they left the ICU, as it would be the effect of a new disease rather than an effect of the care in the ICU itself. This suggests that the ICU patients in our study may have been even less affected by the ICU care.

We chose to use the follow-up data on HRQoL from the 6 months’ measure to get more observations and thereby improve power. A plausible effect that could affect our conclusions is that patients in ICU often do have better HRQoL when examined at 12 months than they had at 6 months [3]. This would lead to more patients having poor HRQoL in the evaluation. The improvements seen at 12 months are most pronounced in the physical dimensions, but the nature of our data means that such an effect has not been overlooked, and we think that this effect is minor and does not alter our conclusions.

Concerning patients lost to follow-up (Figure 1), and who did not respond at all: it is important to stress that one of the symptoms of PTSD is avoidance, and such patients may avoid any reminders of participation. Thus we cannot exclude that many patients with PTSD-related symptoms did not answer the questionnaire and concomitantly may also add to the number of patients having a low HRQoL after critical illness.

Other limitations of the study include the well-known risk of underreporting psychological and/or psychiatry-related problems when using questionnaires. Further, generalizability issues due to study specifics, such as an upper age limit, and cultural and economic differences in other regions of the world, may hamper wider comparisons.

## Conclusions

These data show that, after stratifying for coexisting conditions and adjusting for differences in age and sex, SF-36 HRQoL scale scores between ICU patients and a normal population are insignificant. However, as a new and important finding in this study, we found that in a subgroup of patients with low scores, the mental dimensions were reduced in up to 22% (*n* = 51) and seen in patients with more than one comorbidity. This group was characterized by more often being male, single, on sick leave before admission to ICU, and also of having a short survival time after discharge from the ICU. This group should be an important target for future interventions.

## Key messages

• After stratification for coexisting disease and adjusting for age and sex, no significant differences in health-related quality of life were seen between the ICU group and the reference group 6 months after discharge from the ICU and hospital.

• About 22% of the ICU patients had SF-36 scores below the reference group’s lower level (-2 SD). This effect was seen mainly in the mental dimensions.

• Characteristics for ICU patients with low HRQoL (-2 SD or more) were being male, single, on sick leave before admission to the ICU, and having a short survival time after discharge from the ICU.

## Abbreviations

APACHE II: Acute physiology and chronic health evaluation score; BP: Bodily pain; GH: General health; HRQoL: Health-related quality of life; ICU: Intensive care unit; LIVA: Livskvalitet Intensivvård (Swedish); MH: Mental health; PF: Physical functioning; RE: Role emotional; RP: Role physical; SD: Standard deviation; SF: Social functioning; SF-36: The short form 36 health survey; SPSS: Statistical package for the social sciences; UK: United Kingdom; VT: Vitality.

## Competing interests

The authors declare that they have no competing interests.

## Authors’ contributions

LO designed the study, performed and interpreted the data analysis, and drafted the manuscript; MF performed and interpreted the data analysis; FS designed the study, performed the data analysis, and drafted the manuscript; and MK and SW revised the manuscript. All authors read and approved the final manuscript.
